# Evaluation of Cluster Algorithms for Radar-Based Object Recognition in Autonomous and Assisted Driving

**DOI:** 10.3390/s24227219

**Published:** 2024-11-12

**Authors:** Daniel Carvalho de Ramos, Lucas Reksua Ferreira, Max Mauro Dias Santos, Evandro Leonardo Silva Teixeira, Leopoldo Rideki Yoshioka, João Francisco Justo, Asad Waqar Malik

**Affiliations:** 1 Department of Electronic, Federal Technological University of Paraná, Ponta Grossa 84017-220, PR, Brazil; danielcarvalhoramos@alunos.utfpr.edu.br (D.C.d.R.); lucasreksua@alunos.utfpr.edu.br (L.R.F.); 2 Faculty of Science and Technology in Engineering, University of Brasilia, Gama 72444-240, DF, Brazil; evandroleonardo@unb.br; 3 Polytechnic School, University of São Paulo, São Paulo 05508-010, SP, Brazil; leopoldo.yoshioka@usp.br (L.R.Y.); joao.justo@usp.br (J.F.J.); 4 Department of Electrical and Computer Engineering, Mississippi State University, 406 Hardy Road 216 Simrall Hall Mississippi State, Starkville, MS 39762, USA; asad.malik@msstate.edu

**Keywords:** perception systems, driving assistance, autonomous vehicles, object identification, cluster algorithms, radar sensor, point clouds, K-Means, Mean-Shift, DBSCAN, automotive applications

## Abstract

Perception systems for assisted driving and autonomy enable the identification and classification of objects through a concentration of sensors installed in vehicles, including Radio Detection and Ranging (RADAR), camera, Light Detection and Ranging (LIDAR), ultrasound, and HD maps. These sensors ensure a reliable and robust navigation system. Radar, in particular, operates with electromagnetic waves and remains effective under a variety of weather conditions. It uses point cloud technology to map the objects in front of you, making it easy to group these points to associate them with real-world objects. Numerous clustering algorithms have been developed and can be integrated into radar systems to identify, investigate, and track objects. In this study, we evaluate several clustering algorithms to determine their suitability for application in automotive radar systems. Our analysis covered a variety of current methods, the mathematical process of these methods, and presented a comparison table between these algorithms, including Hierarchical Clustering, Affinity Propagation Balanced Iterative Reducing and Clustering using Hierarchies (BIRCH), Density-Based Spatial Clustering of Applications with Noise (DBSCAN), Mini-Batch K-Means, K-Means Mean Shift, OPTICS, Spectral Clustering, and Gaussian Mixture. We have found that K-Means, Mean Shift, and DBSCAN are particularly suitable for these applications, based on performance indicators that assess suitability and efficiency. However, DBSCAN shows better performance compared to others. Furthermore, our findings highlight that the choice of radar significantly impacts the effectiveness of these object recognition methods.

## 1. Introduction

The development of advanced driving assistance systems (ADAS), as well as Autonomous Driving (AD), is contingent upon the efficacy of perception systems that facilitate the identification and classification of objects in the vehicle’s surrounding environments [1]. These systems utilize an array of sensors, including Radio Detection and Ranging (RADAR), cameras, LIDAR, ultrasonic sensors, and HD maps, to ensure the reliability and robustness of the navigation framework [2]. Among those systems, radar sensors are particularly noteworthy due to their ability to function under various weather conditions, in order to determine the relative position and speed of neighboring objects [3]. Furthermore, the radar generates a point cloud, as this cloud technology facilitates mapping accurately the shapes of objects, which is crucial for grouping and subsequent object recognition [4,5].

Clustering algorithms play a key role in enhancing the capabilities of radar systems to identify objects [6]. It is important to study and apply clustering algorithms in radar systems, particularly on rainy and cloudy days, as there is more difficulty in identifying and classifying objects [7]. Therefore, despite extensive development of various clustering algorithms, a comprehensive assessment of their suitability is lacking, and applications in automotive radar systems are lacking; this gap is essential for advancing the reliability and efficiency of perception systems in autonomous vehicles. The current automotive radar systems allow one to identify, recognize, and track objects in their field of view using cluster algorithms through the cloud points under any weather condition in real time, such as pedestrians, cyclists, animals, other vehicles, and infrastructure elements [7,8].

The radar system is particularly suitable for automotive applications as vehicles are excellent reflectors of electromagnetic waves and, therefore, determine accurately the inter-vehicular distance and speed, as well as the distances to other objects. The radar is extremely important for application in autonomous vehicles since their reliability and multi-functionality make them critical parts in modern ADAS and AD [9,10,11]. The move to higher frequencies from 76 to 81 GHz for radar sensors brought new challenges.

High-resolution automotive radar detects thousands of reflection points in the environment around you, such as vehicles, pedestrians, and traffic. For object classification and recognition, the first step is detecting points that belong to the same object, which must be grouped before further processing [12].

This article aims to show the importance, comparison, and efficiency among clustering algorithms in radar systems and fill the gap by conducting an extensive assessment of multiple groups, as well as test algorithms to determine their applicability to automotive radar systems. Our study examines a range of contemporary clustering methods, including Affinity Propagation, Hierarchical Clustering, BIRCH, DBSCAN, K-Means, Mini-Batch K-Means, Mean Shift, OPTICS, Spectral Clustering, and Gaussian Mixture. Through our analysis, we have identified K-Means, Mean Shift, and DBSCAN as being particularly well-suited for these applications, based on performance indicators assessing their suitability and efficiency.

Our contributions include a detailed comparative analysis of these clustering algorithms, providing insights into their performance and effectiveness in automotive applications. We highlight the significant impact of radar sensor choice on the effectiveness of object recognition methods, underscoring the importance of selecting appropriate radar systems. By enhancing the understanding of the interplay between clustering algorithms and radar data, this study puts forward the challenges for the development of more reliable and robust perception systems for Autonomous Driving and driving assistance technologies.

This work carried out a study of clustering algorithms that are currently used for object recognition in automotive applications. The following contributions were made:

- Comprehensive Evaluation of Clustering Algorithms: This paper provides an extensive analysis of various clustering algorithms, including Affinity Propagation, Hierarchical Clustering, BIRCH, DBSCAN, K-Means, Mini-Batch K-Means, Mean Shift, OPTICS, Spectral Clustering, and Gaussian Blending, specifically for their application in automotive radar systems.
- Identification of Suitable Algorithms: This study identifies K-Means, Mean Shift, and DBSCAN as the most suitable clustering algorithms for radar-based object identification, recognition, and tracking in autonomous vehicles.
- Performance Indicators for Suitability and Efficiency: This paper uses specific performance indicators to assess and compare the suitability and efficiency of the evaluated clustering algorithms, providing a clear metric for their effectiveness in automotive applications.
- Impact of Radar Choice on Object Recognition: This research highlights the significant influence of radar sensor choice on the performance of object recognition methods, emphasizing the importance of selecting appropriate radar systems for optimal results.
- Contribution to Autonomous Driving Technology: By enhancing the understanding of how different clustering algorithms perform in conjunction with radar systems, this study contributes to the development of more reliable and robust perception systems for autonomous vehicles and driving assistance technologies.

This article is organized as follows: Section 2 presents technical aspects of automotive radar systems describing the signal processing, cloud generation TICC model-based clustering method, object detection approach, and ECU radar technology. Section 3 overviews the math description of the cluster and the main algorithms available. Section 4 contains the application of clustering in automotive radar for object recognition. Section 5 presents the comparison analysis of clusters for automotive radar applications such as in the ADAS system. Finally, Section 6 summarizes the contributions.

## 2. The Automotive Radar System

Automotive radar systems are integral components of Advanced Driver Assistance Systems (ADASs), providing critical data for object detection, tracking, and classification. These systems operate at various ranges—short, medium, and long—and utilize different antenna configurations to meet the specific requirements of various ADAS functionalities. The ECU-Radar consists of two main units: the radar sensor (1), which can identify objects in a Field and View and give information in a point cloud format to the radar controller (2), where there are algorithms and strategies to cluster and object detection and recognition. The ECU-Radar can be used by DA or ADAS features such as Adaptive Cruise Control (ACC), Autonomous Emergency Braking (AEB), Forward Collision Warning (FCW), Traffic Jam Assist (TJA), and others. Radars have been used to determine the distance and speed of the vehicle ahead. More recently, a new application has been developed that uses the cloud created from points in the radar’s field of view, as the radar can define clusters to identify objects with the help of a clustering risk.

Antenna configurations play a very important role in radar efficiency systems within Advanced Driver Assistance Systems (ADASs), with single antenna setups offering sufficient capabilities for detecting and tracking vehicles in adjacent lanes or at moderate distances, providing balanced performance across various ADAS functions with a moderate cost and complexity level, albeit with limitations in resolution compared to systems utilizing multiple antennas, whereas multiple antenna configurations provide superior resolution and a wider field of view, thereby enhancing the system’s ability to detect and track multiple objects, leading to improved accuracy and reliability, especially crucial for navigating complex traffic scenarios, although these configurations come with increased cost and design complexity.

In the context of long-range radar (LRR), operating at frequencies typically around 77 GHz with a range of up to 250 m or more, single antenna configurations allow basic long-distance detection and object tracking, sufficient for driving in high speed and effective for maintaining safety in simple driving scenarios. However, they may be less able to distinguish between multiple objects at long distances, while multiple antenna configurations significantly improve target resolution and angular accuracy, crucial for advanced Autonomous Driving functions. They provide superior performance in identifying and tracking multiple objects over long distances, which is essential for safe navigation at high speeds; the integration of radar systems with ADAS offers a comprehensive detection solution that increases vehicle safety and autonomy, where the choice between single and multi-antenna configurations depends on the specific ADAS requirements and trade-offs between cost, complexity, and performance.

Ultimately, radar system selection—whether short, medium, or long-range—profoundly impacts the overall effectiveness of the ADAS, with each range serving distinct purposes, from low-speed maneuvering and collision avoidance to high-speed navigation, and Autonomous Driving, ensuring comprehensive coverage and safety in diverse driving scenarios. Through careful selection and integration of appropriate radar systems, manufacturers can develop ADAS that significantly improve vehicle safety and driver convenience and pave the way for fully Autonomous Driving solutions.

Overall, automotive radar systems are essential to improve vehicle safety by providing reliable detection and tracking of objects in the vehicle’s environment, thereby assisting drivers and enabling advanced Autonomous Driving functionalities.

### 2.1. Radar Signal Processing and Point Cloud Generation

**Figure 1:**
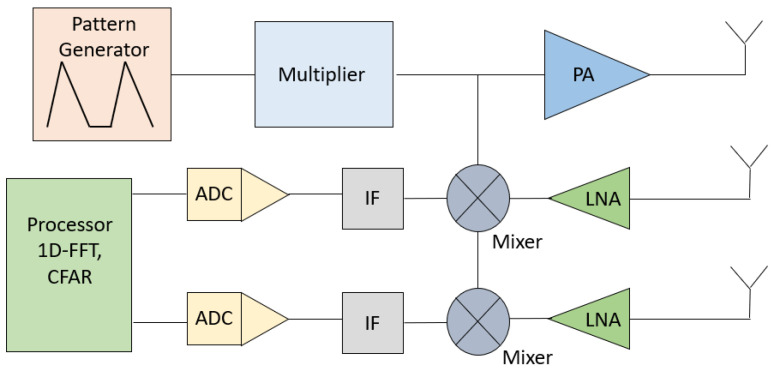
FMCW radar system block diagram.

Radio waves are used to detect objects and determine their range, speed, and angle relative to the vehicle. This process involves several intermediary steps, including signal transmission, reflection, reception, and signal processing, in order to generate point clouds representing the environment around the vehicle. The automotive radar system operates by transmitting Frequency-Modulated Continuous Wave Radar (FMCW) signals, receiving the reflected signals, and processing them to extract beat frequency and Doppler shifts. The processed data are used to generate a point cloud that represents the surrounding environment. The transmitter generates a continuous frequency signal and transmits it, and then the transmitter power is increased by an amplifier. The frequency of the transmitted signal is modified over time and the signal is reflected reaching the receiver, and then the target signal that has been received passes through the mixer and a low noise amplifier (LNA). Therefore, a frequency signal (IF) is obtained and routed to the digital converter (ADC) to be processed in a signal processor, as illustrated in Figure 1.

The radar system transmits an FMCW signal, which is a continuous wave with a frequency that increases linearly over time (a chirp signal) and is given by

(1) $$s\left(t\right)=Acos(2\pi \left(f_{c}t+\frac{B}{2T}t^{2}\right))$$

where *A* is the signal amplitude, $f_{c}$ is the carrier frequency, *B* is the bandwidth, and *T* is the chirp duration.

The transmitted signal reflects off objects and is detected by the radar’s receiving antennas with a time delay τ and a Doppler shift $f_{d}$ due to the relative motion of the objects. The received signal r(t) is described by

(2) $$r\left(t\right)=Acos(2\pi \left(f_{c}\left(t-\tau \right)+\frac{B}{2T}{\left(t-\tau \right)}^{2}\right))$$

where τ is the round-trip time delay and given by $\tau =\frac{2R}{c}$ where *R* is the range to the object and *c* is the speed of the light. The beat frequency is a fundamental concept in radar signal processing. It refers to the frequency difference between transmitted and received signals after they interact with a target. This frequency difference contains crucial information about the target’s range. When a radar signal is transmitted and then reflected off a target and received back at the radar system, changes in the frequency of the received signal occur due to the Doppler effect and the motion of the target relative to the radar system. The transmitted signal is given by $f_{t}$, where is the frequency of the transmitted radar signal. Upon reflecting off a target, the received radar signal’s frequency $f_{r}$ is altered due to the Doppler effect, which depends on the relative movement between the target and the radar system. Then, the beat frequency $f_{b}$ is defined as the difference between the transmitted and received frequencies, as shown in the equation

(3) $$f_{b}=f_{r}-f_{t}$$

The beat frequency can be used to provide a rough estimation of the distance to the target. When the transmitted signal reflects off a target and returns to the radar system, the time delay τ between transmission and reception is related to the target’s distance *R*.

- Time Delay: The time delay τ is twice the time it takes for the radar signal to travel from the radar system to the target and back: $\tau =\frac{2R}{c}$. We consider that *R* is the range of the target and *c* is the speed of light.
- Frequency Modulation: In frequency-modulated continuous-wave (FMCW) radar systems, the beat frequency $f_{b}$ can be related to the time delay τ through the chirp bandwidth *B* and the chirp duration *T*: $f_{b}=\frac{2B}{T}\tau$.
- Range Calculation: Rearranging the equation for τ yields: $R=\frac{f_{b}T_{c}}{4B}$.

The beat frequency represents the frequency difference between transmitted and received signals, providing information about the target’s motion. By analyzing this frequency difference, radar systems can determine the range of detected targets. This calculation is fundamental to radar-based distance measurement and plays a vital role in various applications, including automotive radar systems for collision avoidance, adaptive cruise control, and object detection.

### 2.2. Relative Distance and Speed Calculations

The radar system samples the received signals to generate point clouds and the shape represents a detected object with its range *R*, azimuth angle θ, elevation angle ϕ, and radial velocity $v_{r}$. Given two radar detection events at times $t_{1}$ and $t_{2}$ with positions $\left(R_{1},\theta_{1},\phi_{1}\right)$ and $\left(R_{2},\theta_{2},\phi_{2}\right)$, the relative distance *D* and relative speed $v_{r}$ can be calculated as follows:

- Relative Distance: assuming that the radar coordinate system gives the relative distance *D* between two points such as
  (4) $$D=\sqrt{{\left(R_{2}cos\left(\theta_{2}\right)-R_{1}cos\left(\theta_{1}\right)\right)}^{2}+{\left(R_{2}sin\left(\theta_{2}\right)-R_{1}sin\left(\theta_{1}\right)\right)}^{2}}$$
- Relative Speed: the relative speed $v_{r}$ between the two detection events can be calculated as
  (5) $$v_{r}=\frac{R_{2}-R_{1}}{t_{2}-t_{1}}$$

### 2.3. Object Recognition Through Clustering

Object recognition through clustering is a vital aspect of radar-based perception systems in automotive applications. Clustering algorithms group together radar data points that belong to the same physical object, enabling the identification and tracking of objects such as vehicles, pedestrians, and obstacles. Once the clustering algorithm assigns data points to clusters, various analyses are performed on the clusters to identify and classify objects, as follows:

- Centroid Analysis: The centroid of each cluster represents a potential object. Additional analysis can refine object properties such as size, velocity, and heading.
- Shape Analysis: Analyzing the shape and size of clusters can help distinguish between different types of objects (e.g., vehicles, pedestrians).
- Temporal Analysis: Tracking the movement of clusters over time allows for object trajectory estimation and prediction.
- Statistical Analysis: Assessing cluster properties such as density, spread, and coherence can provide insights into the reliability of object detection.

Once clustering is performed, each cluster represents a distant object and can be calculated as follows:

- Centroid: The average disposition of all cluster points, representing the object’s position
  (6) $$\mu_{i}=\frac{1}{|C_{i}|}\sum_{x\in C_{i}}x$$
- Extent: The spatial spread of the points, representing the object’s size
  (7) $$\sigma_{i}=\sqrt{\frac{1}{|C_{i}|}\sum_{x\in C_{i}}{∥x-\mu_{i}∥}^{2}}$$
- Velocity: The average of all cluster points, representing the object’s speed and direction
  (8) $$v_{obj}=\frac{1}{|C_{i}|}\sum_{x\in C_{i}}v_{r}$$

As we mentioned before, this clustering allows the radar system to recognize and track multiple objects, providing crucial data for driving assistance and Autonomous Driving to make informed decisions for vehicle safety and navigation.

### 2.4. The Radar Measurement Range Technologies

**Figure 2:**
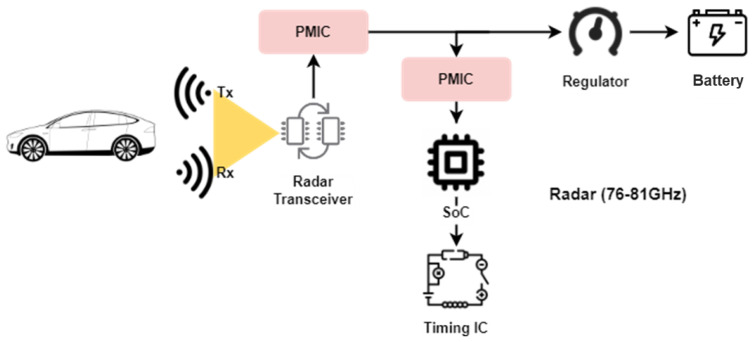
Basic topology of a radar system.

The basic topology of radar includes one or more Monolithic Microwave-Integrated Circuit (MMIC) [13] radar transceivers, which are the sort of integrated circuit device that work out at microwave frequencies (from 300 MHz to 300 GHz), such as the AWR1243, 76-to-81 GHz High-Performance Automotive MMIC from Texas Instruments connected to a performance processing unit (MCU or SoC), shown in Figure 2.

**Figure 3:**
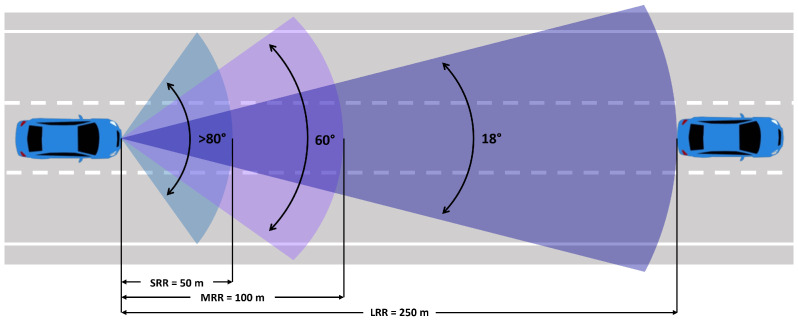
Radar measurement range classification: Short Range Radar (SRR)/Middle Range Radar (MRR)/Long Range Radar (LRR).

According to the International Union of Telecommunication Functions, automotive radars are classified into two categories. Category 1 includes systems of comfort functions, which allow for more stable driving. This category includes adaptive cruise control (ACC) and collision avoidance (AC) systems for an active safety range of up to 250 m. Category 2 defines high-resolution sensors for applications such as blind spot detection, lane change assist, and threat warning. The range is smaller than Category 1, with a maximum distance of 50 m. Radars are classified according to their measurement range, as shown in Figure 3.

With advances in high-frequency integrated circuits (ICs) along with Monolithic Microwave-Integrated Circuits (MMICs), radars (FMCW) can be used in diverse applications [14]. Of the four main frequency bands used in radar systems, two are present in the K band (near 24 GHz) and two are in the E band (between 76 and 81 GHz) [15].

For static objects, the semantic segmentation in the environment generated by the radar reading is performed by a convolutional neural network, and for dynamic objects a new neural network architecture is used to segment recurring instances in radar point clouds of moving objects [16]. After this process of capturing static and dynamic objects, they are returned to the radar targets in this process of the spatial region of probability classes that are assigned to each cell of the note. In a fusion step, the results of both are brought together into a single point cloud.

In the recently developed multiple inputs multiple outputs (MIMO) radar system, which is a multi-antenna radar system, with transmitting antenna functioning independently, receiving antennas can measure these signals [17]. Since the wave forms are different, the echo signals are reassigned to a single transmitter. In this way, it is possible to obtain a virtual field of K-N elements, from a field of antennas of N transmitters with a field of K receivers, the obtained field can be expanded to a virtual aperture size [18,19].

When the radars read the environment, they generate a point cloud, which is defined as a set of four dimensions [20]:

(9) $$Point=\left\{p_{i}|i=1,2,\ldots ,n\right\},\;n\in N$$

where n represents the number of targets hit by the radar, and each point $p_{i}=\left(x,y,v_{r},\sigma \right)$, with (x,y) coordinates and $v_{r}$ is the compensated Doppler velocity, and σ is the radar crossed by section value [7].

### 2.5. The ECU Radar Technology

The ECU radar system plays a critical role in ADAS by processing data from radar sensors and making real-time decisions to enhance vehicle safety and automation. This system typically consists of a microcontroller with a real-time operating system (RTOS), and radar sensors, each component fulfilling a specific function to support various ADAS features.

The radar sensor works by emitting radio waves, thus measuring the time interval waves take to return from objects. By analyzing these signals, the radar sensor can determine the speed, distance, and angle of objects relative to the vehicle. The main components are the transmitter, receiver, signal processor, microcontroller, power, and communications modules.

The Real-Time Operating System (RTOS) manages the execution of multiple tasks, ensuring that radar data are processed within strict timing constraints. This is critical for real-time applications like ADAS and the key features of deterministic scheduling, multitasking, and inter-task communication.

The ECU radar system, comprising a microcontroller RTOS and radar sensor, forms the backbone of many ADAS features, enabling real-time processing and decision-making to enhance vehicle safety and automation. By leveraging the strengths of each component, this integrated system provides robust and reliable assistance to drivers, paving the way for advanced Autonomous Driving capabilities.

**Figure 4:**
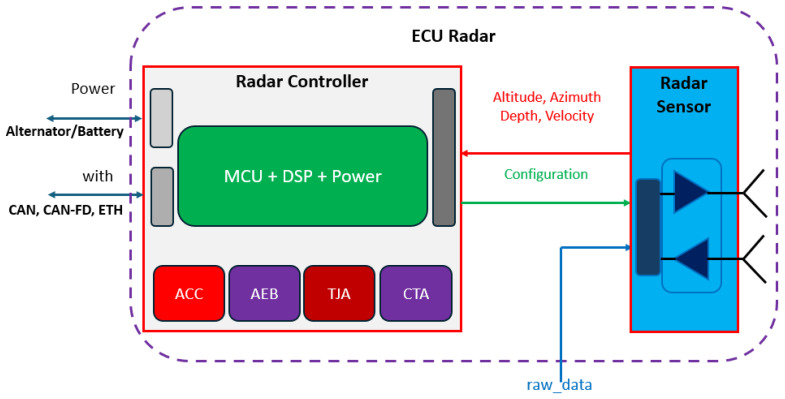
Architecture of the Automotive ECU-Radar with its components, technologies, and applications enabled for DA features.

The ECU-Radar to be designed should comprise an architecture and technologies that meet the requirements of the DA system, pursuing flexibility, accuracy, and robustness in compliance with standards and regulations. The entire architecture of the components and its integration is shown in Figure 4.

The ECU-Radar shall have the states of configuration, calibration, and application defined by the user using the appropriate tool. The manual shall be provided easily and accurately. According to Figure 4, the ECU-Radar comprises two main integrated components. The radar sensor operates based on the principle of RADAR (Radio Detection and Ranging). They emit radio waves in specific frequencies and analyze the reflected signals to determine the distance, speed, and direction of objects in the vehicle neighborhood. The radar controller with ADAS features is a specialized component within a vehicle’s electronic system that manages radar sensors and integrates them with the vehicle’s safety and driver assistance functions. The radar controller is composed of a microcontroller (MCU), Digital Signal Processing (DSP), plus Power Supply.

The Electronic Control Unit (ECU) for radar-based ADASs (Advanced Driver Assistance Systems) performs sophisticated information processing to ensure accurate and real-time object detection and classification. This processing involves several stages, from data acquisition to decision-making, with clustering algorithms playing a crucial role in interpreting radar data.

1. Data acquisition through the radar sensor output;
2. Preprocessing the signal filtering and noise reduction;
3. Clustering algorithm to group individual radar data points into clusters that represent distinct objects in the environment;
4. Object detection and classification with feature extraction and object classification;
5. Multi-Object Tracking (MOT) through Kalman filters or more advanced techniques like particle filters track the detected objects over time;
6. Decision-making to make real-time decisions and control the vehicle.

The ECU radar system with clustering algorithms and object detection processes radar data through several stages, including data acquisition, preprocessing, clustering, object detection, classification, tracking, and decision-making. Each stage involves complex mathematical models and algorithms to ensure accurate and real-time perception, enabling the reliable and safe operation of ADAS features in autonomous vehicles.

**Figure 5:**
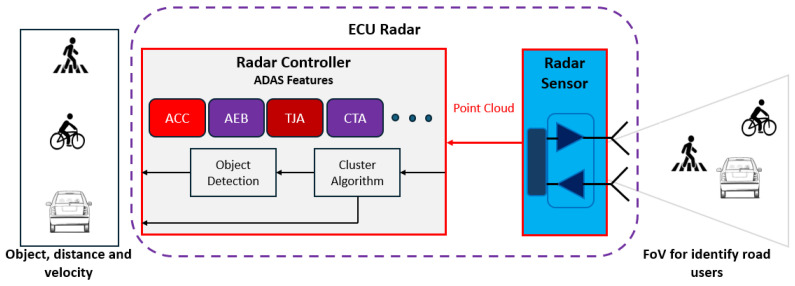
Information processing of automotive ECU-Radar.

Figure 5 shows how the ECU-Radar should process information from sensor detection to the DA application. The radar sensor has to identify the road used and gives information in a point cloud format. The radar controller has cluster and object detection algorithms to identify and classify objects to be used by DA features.

After all these explanations, we are ready to understand more about singular cluster algorithms to identify the best one to be deployed on an automotive radar system. First, we identified those available in the literature, and next, we figured out those that fit the radar. Finally, we used three more automotive radar suitable for COTS.

## 3. The Main Cluster Algorithms

Clustering algorithms are a class of machine learning techniques that group a set of points or data into clusters based on elementary similarity. These algorithms detect patterns and configurations in the data to ensure that the points that are within that particular cluster are similar to each other and different from points that are in other clusters. Clustering is a type of unsupervised learning, meaning that it does not rely on predefined labels for the data points.

Now, in a dataset, a cluster group is used to set up each point in that data group into a particular group. Data points that are part of that given group must have similar characteristics and properties, in the same way that data points that are in other groups must have different properties [19].

A mathematical description of clustering involves defining the method of grouping a set of data points into clusters, such that points in the same cluster fit more closely to each other than those in other clusters. Here is a detailed mathematical description of clustering and some popular clustering algorithms:

Basic concepts might be defined by the following:

1. Data Points: Consider $X=\{x_{1},x_{2},x_{3},...,x_{n}\}$ a set of points (data), where each $x_{i}$ is a vector in a ${ℜ}^{d}$. Considering a set of *n* objects
  (10) $$X=\{x_{1},x_{2},x_{3},...,x_{n}\}$$
2. Clusters: A clustering is a partition of the dataset *X* into $C_{i}$ clusters $C=\{c_{1},c_{2},...,c_{n}\}$ such that
  (11) $$\overset{k}{\underset{i=1}{⋃}}C_{i}=X\;\mathrm{and}\;C_{i}\cap C_{j}=\emptyset \;\mathrm{for}\;i\neq j$$
3. Centroid: The centroid $\mu_{j}$ of a cluster $C_{j}$ is the mean position of all the points in the cluster
  (12) $$\mu_{j}=\frac{1}{|C_{j}|}\sum_{x\in C_{j}}x$$
4. Distance metric: A function $d={ℜ}^{d}\times {ℜ}^{d}\to ℜ$ that defines the similarity between data points. Consider the Euclidean distance
  (13) $$d\left(x,y\right)={∥x-y∥}_{2}=\sqrt{\sum_{i=1}^{d}{\left(x_{i}-y_{i}\right)}^{2}}$$

These mathematical formulations and algorithmic steps provide a basis for understanding clustering and its application in various domains, including automotive radar systems.

Several clustering algorithms have been widely used recently, such as Hierarchical Clustering, Affinity Propagation, DBSCAN, BIRCH, K-Means, Mini-Batch K-Means, OPTICS, Mean Shift, Mixture of Gaussians, and Spectral Clustering. They all have specific characteristics, which may be more suitable for certain specific applications that require data grouping. A detailed description of several clustering algorithms, commonly applied in automotive radar systems, is presented below. These clustering algorithms offer various strengths and weaknesses, making them suitable for different scenarios in automotive radar systems. The choice of an algorithm depends on factors such as the density and distribution of data points, computational efficiency, and the specific requirements of the Driving Assistance and Autonomous Driving question.

### 3.1. Affinity Propagation

Affinity Propagation contains a low error and is fast and flexible, which has the advantage of taking similarity measurements between pairs of data points as input parameters while considering other data points as potential examples. Data points exchange messages of real value, until the set with high-quality samples starts to appear [20]. The algorithm then goes through several iterations until reaching convergence based on a pre-defined criterion. Each iteration has two message-passing steps. The first step is the passive r(i,k), the passive takes into account how suitable the point *k* is to serve as an example for the point *i*, comparing it with the other potential examples for the point *i*. Responsibility is sent from data point *i* to candidate example point k. The second step is the calculation of availability, which is how much the availability a(i,k) equates to how appropriate it would be for point *i* to choose point k as its reference point; considering the support of other points, this point *k* is an example. The availability is sent from candidate exemplar point k to point *i*. When calculating responsibilities, the algorithm uses the original similarity and the availability calculated in the previous iteration. In the first iteration, these variables are set and initialized to zero [21].

The algorithm is based on the following equations:

(14) $$a(k,k)=\sum_{i^{\prime }\;such\;that\;i^{\prime }\neq k}max\left\{0,r\left(i^{\prime },k\right)\right\}$$

where self-availability a(k,k) is defined as the sum of positive responsibilities that the exemplary candidate *k* receives from other points.

(15) $$r(i,k)\leftarrow s(i,k)=\sum_{k^{\prime }\;such\;that\;k^{\prime }\neq k}max\left\{a\left(i,k^{\prime }\right)+s\left(i,k^{\prime }\right)\right\}$$

where the responsibility r(i,k) reflects the accumulated evidence s(i,k) of how well point *k* is suitable to serve as an example for point *i*.

(16) $$a(i,k)\leftarrow min\{0,r(k,k)\}+\sum_{i^{\prime }\;such\;that\;i^{\prime }\notin \left\{i,k\right\}}max\left\{0,r\left(i^{\prime },k\right)\right\}$$

where availability a(i,k) is defined as the self-responsibility r(k,k) plus the sum of the positive responsibilities that the exemplary candidate *k* receives from other points [20].

### 3.2. Hierarchical Clustering

The Hierarchical Clustering algorithms differ from partition-based ones in that they build a binary merge tree, which falls into two types of categories, top-down and bottom-up [22]. Ascending clusters initially consider each data point as a single cluster and repeat this process until all the clusters come together and form just a single cluster with all the data. This process generates a graphical representation of a tree that incorporates the nodes in the plane, which is called deprogramming [23,24].

To understand this algorithm, the distance metrics must be defined. First, the Euclidean distance is the shortest distance between two points in any dimension.

(17) $${∥a-b∥}_{2}=\sqrt{\sum_{i}{\left(a_{i}-b_{i}\right)}^{2}}$$

where $a_{i}$ and $b_{i}$ represent, respectively, the characteristic of individual *a* and *b*, and *i* is the number of plots in the sample. The square Euclidean distance is described by

(18) $${∥a-b∥}_{2}^{2}=\sqrt{\sum_{i}{\left(a_{i}-b_{i}\right)}^{2}}$$

On the other hand, the sum of the absolute differences in coordinates between two points is called the Manhattan distance

(19) $${∥a-b∥}_{1}=\sum_{i}|a_{i}-b_{i}|$$

Moreover, the following equation provides the mathematical definition of the maximum distance between two points, as follows:

(20) $${∥a-b∥}_{\infty }=\max_{i}\left|a_{i}-b_{i}\right|$$

After choosing the definition of distance, it is important to choose the connection criteria, as simple, complete, or average connection.

In Hierarchical Clustering, defining the number of clusters is unnecessary, as one can select the number of clusters that seems the best one as the tree is being built. Furthermore, in this algorithm, all distance analysis works well, unlike the other algorithms presented in this work, where the selection of distance analysis is a decisive factor.

An interesting system for Hierarchical Clustering is when the data have an underlying hierarchical structure and such hierarchy must be retrieved, while other clustering algorithms cannot determine such hierarchy. However, these advantages mean that this algorithm has a time complexity of O (n³) with lower efficiency, unlike the linear complexity of other algorithms presented in this work, such as K-Means and GMM [23].

### 3.3. BIRCH

BIRCH is a clustering algorithm with a Hierarchical Clustering methodology, which has been designed to work with large numerical datasets. It uses the idea of clustering features (CF) [25]. The CF is a three-dimensional vector that has information about the objects, defined by

(21) CF=<n,LS,SS

with *n* being the number of points in a cluster, LS defined as the sum of *n* points and SS equal to the squared sum of *n* points.

The CF has the required information for decision-making of the BIRCH algorithm, which is taken from a reading of the dataset. Through the CF, an initial CF tree is built, which stores the CF for a hierarchical grouping, which can be understood as a data compression trying to preserve its characteristics.

The CF characteristics are additive; that is, if we have two disjoint clusters $C_{1}$ and $C_{2}$, which have $CF_{1}$ and $CF_{2}$, respectively, a new cluster composed of the junction of $C_{1}$ and $C_{2}$ is simply $CF_{1}+CF_{2}$.

The BIRCH algorithm applies a multifaceted technique with a single data reading, and if necessary, the algorithm makes one or two additional readings to improve the quality of the result [25,26].

Given *n* points $x_{i}$, in a d-dimensional space, this group of points can be defined as being a cluster, in which the following parameters identify it:

(22) $$x_{0}=\frac{1}{n}\sum_{i=1}^{n}x_{i}$$

where $x_{0}$ is the centroid that is given by Equation (22), *R* is the radius which is given by Equation (23), and *D* is the diameter which is given by Equation (24).

(23) $$R=\sqrt{\frac{1}{n}\sum_{i=1}^{n}{\left(x_{i}-x_{0}\right)}^{2}}$$

(24) $$D=\sqrt{\frac{1}{n(n-1)}\sum_{i=1}^{n}\sum_{j=1}^{n}{\left(x_{i}-x_{j}\right)}^{2}}$$

If we consider *R* as the average distance from the centroid to the objects in the cluster, *D* is defined as the average distance between pairs within a cluster. They measure the data’s spread (or concentration) around the cluster’s centroid. The BIRCH algorithm has three parameters:

- Limit: it is the largest data value that a subcluster has in the leaf node of the CF tree.
- Branch Factor: maximum CF sub-clusters value at each node.
- Number of clusters: it is the number of clusters that return after the entire BIRCH algorithm process is completed.

### 3.4. DBSCAN

The algorithm Density-Based Spatial Clustering of Noise Application (DBSCAN) is a density-based non-parametric clustering algorithm similar to mean deviation. It is a very effective method for identifying clusters of arbitrary shapes and different sizes, as it identifies and separates noise from data without any preliminary information about the group [27]. The DBSCAN algorithm only requires two factors: the size of the radius epsilon, the size of the radius ϵ, and also the number of minimum elements in the neighborhood (MinPts). These parameters are explained below [28].

**Figure 6:**
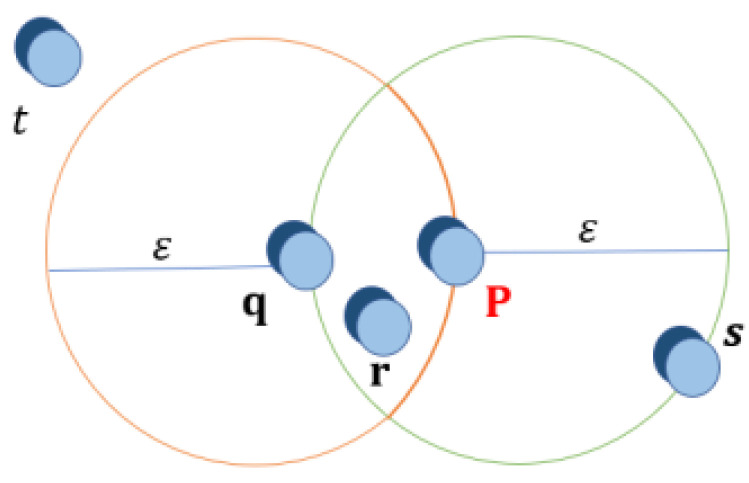
Neighborhood of a point: each point in a cluster has its neighborhood with a certain radius that contains at least a certain number of points.

**Figure 7:**
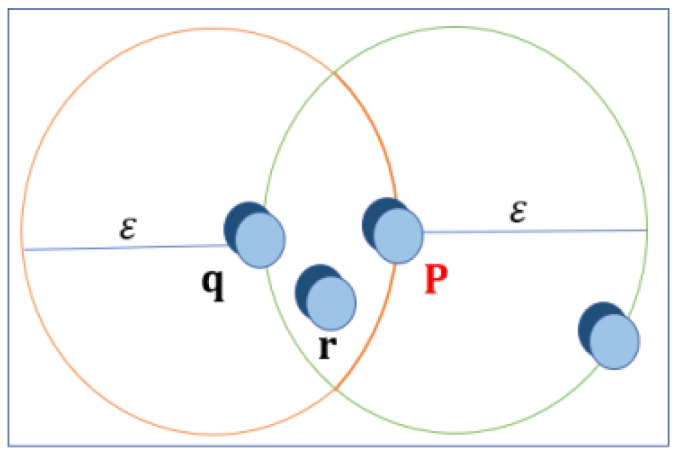
Direct Density Reach is when the object *p* is directly reachable by the density of object *q*, when *p* is ϵ-neighborhood of *q*, and *q* is a midpoint.

First, let us define ϵ-neighborhood stands for a point that can be viewed in Figure 6 and the central point is defined when the ϵ-neighborhood of an object p contains a minimum number, MinPts, of objects. Then, the p object is called a center point. The edge points are where the ϵ-neighborhood of an object contains less than MinPts, but it has a center point, so object p is defined as an edge point. Simularly the Direct Density Reach is defined when the object *p* is within the density range of the object *q* while *p* is in the ϵ-neighborhood of *q*, and *q* is a midpoint, as shown in Figure 7.

**Figure 8:**
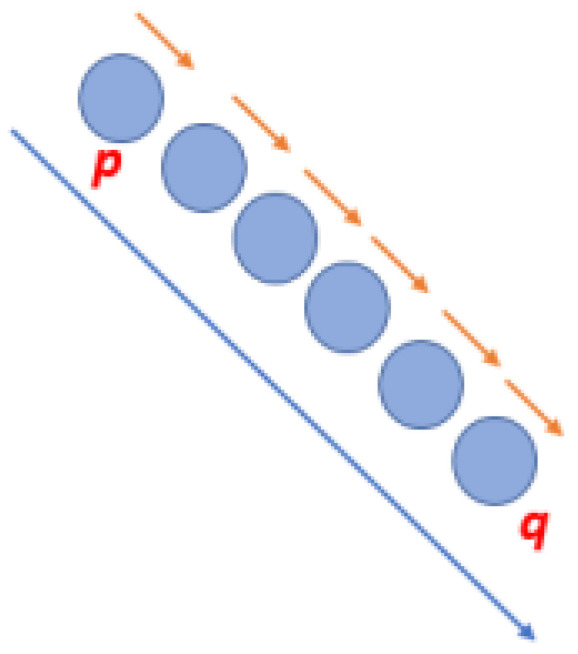
Reach by Density is defined when the object *p* is reachable by the density of object *q*, in a set D, if there is a chain of objects, such that *p* is reachable by density directly from *q* with respect to MinPts.

Reach by Density is shown in Figure 8.

Density connection happens when an object *p* is connected by the density of object *q*, in a set D, if there is an object where both *p* and *q* are reachable by object density.

The DBSCAN cluster is defined when a set of database points D and a cluster C with respect to ϵ and MinPts is a non-empty subset of D that satisfies the following conditions:

1. ∀p,q: if *p*ϵ*C* and *q* is reachable by density from *p* with respect to and MinPts, then *q*ϵ*C*.

2. ∀p,q: if *p*ϵ*C*: *p* is connected by density to q with respect to ϵ and MinPts.

Consider $C_{1},C_{2},\ldots ,C_{k}$ the database clusters D against the configurations ϵ and MinPts$i_{1},i_{2},\ldots ,i_{k}$, then the noise can be described as the set of points in the database D that does not belong to any group $C_{i}$, so the noise is as follows:

(25) $$\left\{p\in D|\forall i:p\notin C_{i}\right\}$$

**Figure 9:**
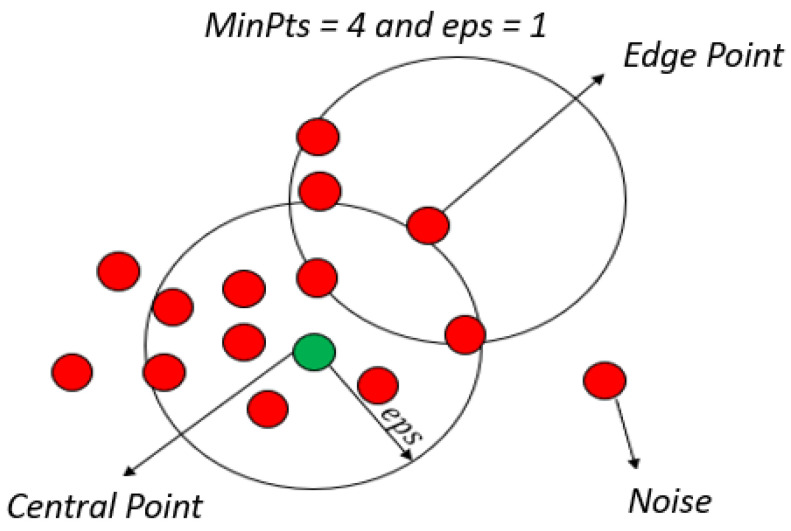
The parameters of DBSCAN.

Figure 9 presents the parameters in the DBSCAN algorithm, center point, edge point, and noise.

### 3.5. K-Means

The K-Means algorithm belongs to the group of partitional clustering algorithms, which have been widely used in many applications [29], and the objective function most used for metric spaces in partitional methods [30]. It has been one of the most-used clustering methods, as it is simple, easy to implement, and has low computational complexity [29,31].

**Figure 10:**
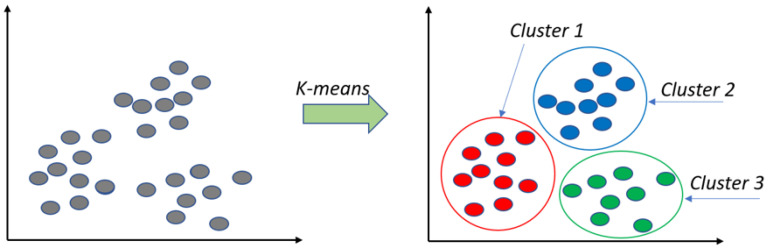
Point clustering using K-Means algorithm.

K-Means is widely used due to its simple and fast-converging algorithm [32]. However, the K value of the clusters must be provided in advance as it directly affects the convergence results [33]. The Point clustering using K-Means algorithm can be viewed in Figure 10.

The most-used objective function for metric spaces in partitional methods is the quadratic error [34], given by

(26) $$E=\sum_{j=1}^{k}\sum_{x\in C_{i}}{∥p-m_{i}∥}^{2},\;\;\mathrm{for}\;k\in \left(1,n\right)$$

### 3.6. Mini Batch K-Means

The Mini Batch K-Means algorithm uses smaller groups of randomly selected data, which remains in memory and the defined iterations constantly update the cluster until convergence. Each small group uses a combination of values and data to help update its cluster, a learning rate is applied to help account for the number of iterations. The learning rate has been defined as the inverse of the number of data assigned to the cluster, such that the iterations increase, convergence is blocked, and there is no change in the groups formed, after several consecutive iterations [35].

The algorithm randomly chooses small batches from the dataset for each iteration. Data from each batch are assigned to clusters depending on the locations of the cluster centroid positions in the previous iteration. As a result, it updates the cluster centroid locations based on the new graph points.

Mini Batch K-Means presents results that are considerably different from K-Means. As the number of clusters and the amount of data increase, more intensive computational resources are required. Therefore, for large amounts of data and clusters, the final result between K-Means and Mini Batch K-Means shows a reduced similarity in the clusters [35].

### 3.7. Mean Shift

Mean Shift algorithm works based on a sliding window, whose objective is to find dense areas of data points. It is a centroid-based algorithm, aiming to find the midpoints of each group, which updates the candidates to midpoints, such that they are the average of the points within the sliding window. In a post-processing stage, these candidate windows are filtered, in order to eliminate duplicates, thus forming a final set of center points and their corresponding groups [36].

The processing starts with a sliding window centered on a point *C*, which is circular and the point *C* is chosen in a requested manner having a nucleus of radius *r*. Mean Shift aims to move this kernel iteratively to a region of greater density until convergence. After each iteration, the sliding window is shifted to a region of higher density, therefore moving the center point to the middle of the points within the window. The density within the sliding window is proportional to its number of points. When moving to the midpoint in the window, it gradually moves to where it contains the greatest number of points.

The sliding window continues to update based on the average until it is no longer possible to accommodate any more points within the kernel. It is performed with several sliding windows, until all the points are within a single window. When there is an overlap between sliding windows, the window containing more points is preserved. As a result, data points are grouped taking into account the sliding window in which they reside.

**Figure 11:**
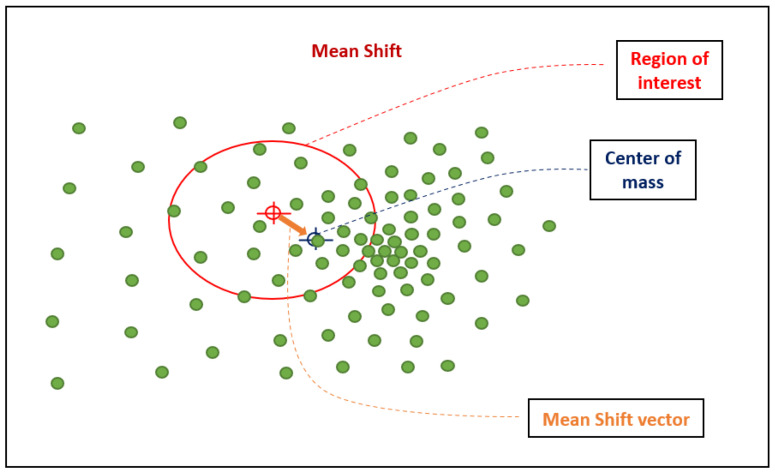
Mean Shift algorithm parameters.

Unlike K-Means clustering, there is no need to define the number of clusters as the average displacement automatically finds it, which is a major advantage. The fact that the centers of the clusters converge to the points of maximum density is also very desirable as it fits well in a naturally data-driven sense. The disadvantage is that defining the size/radius of window “r” may not be trivial [36]. Figure 11 shows the Mean Shift algorithm parameters, such as region of interest, the center of mass, and Mean Shift vector.

### 3.8. OPTICS

The OPTICS algorithm is similar to DBSCAN. It creates an accessibility graph, which is used to extract clusters [37]. To understand how OPTICS works, it is necessary to know how the DBSCAN algorithm works, mainly the parameters that are considered and the main difference between center points and limits.

The core distance is the minimum epsilon to make a point distinct from a center point, given finite MinPts parameters.

The accessibility distance of an object p relative to another object o is the smallest distance of o if o is a central object. Nor can it be less than the distance from the core.

Although the MinPts parameter is used in these calculations, the idea is that it would not have much of an impact because all distances would scale at approximately the same rate. These definitions are used to create an accessibility graph, which is used to extract the clusters. First, it starts by calculating the distances from the core at all data points in the set. Then, it loops through the entire dataset, and updates the accessibility distances, processing each point just once. There is only an update accessibility distances for points that need to be improved and have not yet been processed. The next data point chosen to process is the one with the closest range distance. This is how the algorithm keeps the clusters close to each other in the output sort [37].

**Figure 12:**
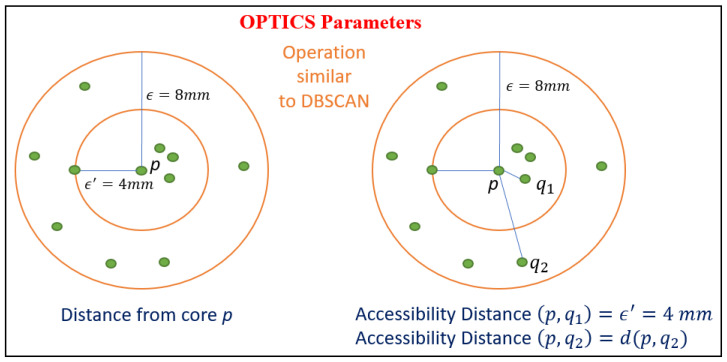
OPTICS algorithm parameters.

Figure 12 shows the optics algorithm parameters, such as distance from core and accessibility distance.

### 3.9. Spectral Clustering

Spectral Clustering is defined as increasing cluster behavior. The implementation consists of constructing the similarity graph, which is built as an adjacency matrix (represented by A). This matrix is constructed with the epsilon-Neighborhood graph, an epsilon parameter. As a result, each point is connected to all other points within its epsilon radius. If all distances between any two points are similar in scale, then the distance between the two points are not stored, as no additional information is obtained. In this case, the constructed graph is an undirected and unweighted graph. Another way to construct the graph is K-neighbors, where a parameter k is defined. Therefore, for two vertices *u* and *v*, an edge is directed from *u* to *v* only if *v* is among the k nearest neighbors of *u*. This process leads to the formation of a thoughtful and targeted graph [29].

### 3.10. Mixture of Gaussians

Gaussian Mixture Models (GMMs) is a function comprising several Gaussians, each one identified by

k∈{1,…,K}

The Gaussian *k*, present in the mixture, are composed of the following parameters: a center defined by a media μ, a width that defines a covariance Σ. There is a mixture probability π that defines the size of the Gaussian function, which can be large or small [38].

**Figure 13:**
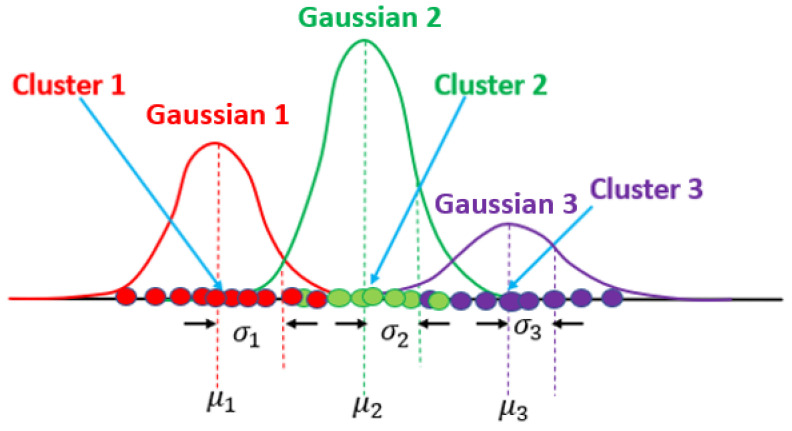
Mixture of Gaussian: three Gaussian functions are illustrated, so K = 3. Each Gaussian explains the data contained in each of the three available clusters.

Figure 13 presents a set of data, arranged in three Gaussians, where each one represents a cluster.

Mixing coefficients are probabilities, which must be constrained by

(27) $$\sum_{k=1}^{K}\pi_{k}=1$$

The parameters must be chosen so that they are as close to the ideal value as possible, which is conducted by ensuring that each Gaussian fits the data points belonging to each cluster, the maximum probability function is used [27], the Gaussian density function is given by

(28) $$N\left(x|\mu ,\sum \right)=\frac{1}{{\left(2\pi \right)}^{\frac{D}{2}}{\left|\sum \right|}^{\frac{1}{2}}}\exp\left(-\frac{1}{2}{\left(x-\mu \right)}^{T}\sum^{-1}\left(x-\mu \right)\right)$$

where *x* represents the data points, *D* the number of dimensions of each data point, μ the average, and Σ the covariance.

As shown at the beginning, the sum of all mixing coefficients π is equal to one. The sum of the probabilities γ over k also gives 1. Thus, λ=N, which allows to solve for π.

In this way, by performing the calculations described in [14], it is possible to obtain

(29) $$\pi_{k}=\frac{\sum_{n=1}^{N}\gamma \left(z_{nk}\right)}{N}$$

(30) $$\mu_{k}^{*}=\frac{\sum_{n=1}^{N}\gamma \left(z_{nk}\right)x_{n}}{\sum_{n=1}^{N}\gamma \left(z_{nk}\right)}$$

(31) $$\sum_{k}^{*}=\frac{\sum_{n=1}^{N}\gamma \left(z_{nk}\right)\left(x_{n}-\mu_{k}\right){\left(x_{n}-\mu_{k}\right)}^{T}}{\sum_{n=1}^{N}\gamma \left(z_{nk}\right)}$$

Table 1 and Table 2 summarize a comparison among all those algorithms.

## 4. Application of Clustering in Automotive Radar for Object Recognition

**Figure 14:**
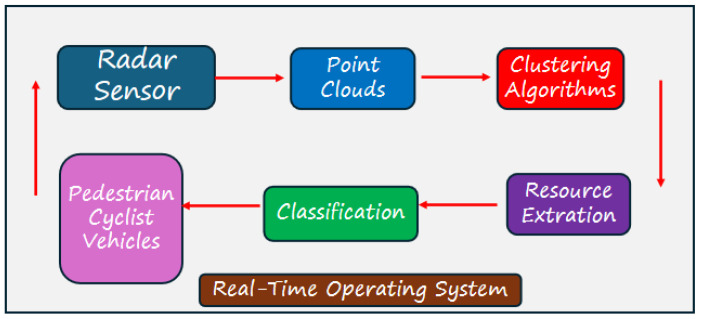
Automotive radar process from point cloud to cluster and object detection and recognition.

The clustering algorithm from point clouds, acquired from the radar, enables one to set the objects’ shape in a field of view to identify, recognize, and track them. The radar is a potential sensor for those applications due to its robustness and reliability to work in different weather conditions. Figure 14 shows how clustering theory is used in an automotive radar system, in driving assistance applications, or in independent vehicles. When the vehicle is moving, the cycle repeats itself and the radar always reads the environment so that the algorithm recognizes objects in the environment. The idea for creating this figure was applied in the article Multi-Stage Clustering Framework for Automotive Radar Data [11].

**Figure 15:**
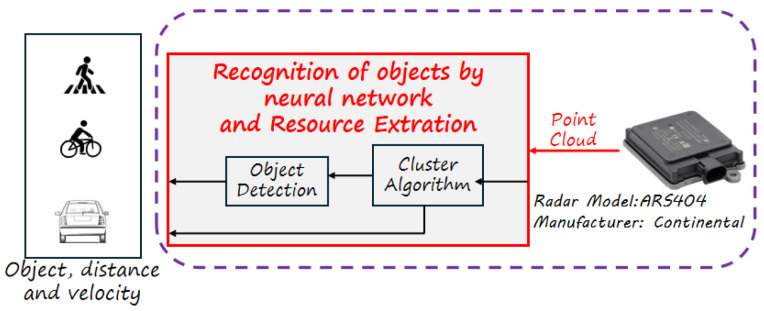
Process for applying clustering radar system.

In the block that deals with feature extraction, we can use numerous tools that recognize objects, such as YOLOv4 and YOLOv5, which are great training networks [39,40]. In Figure 15, the point clouds generated by the radar, to which the clustering algorithm would be applied, and subsequently an object recognition technique via neural networks would be used, resulting in identified and classified objects such as pedestrians, vehicles, cyclists, and trucks. This process was applied in the article Multi-Stage Clustering Framework for Automotive Radar Data [11].

The radar system is based on ARS 404-21© model from Continental Automotive Company. The ARS 404-21 sensor independently measures distance and velocity to non-reflective objects based on the Doppler principle, utilizing Frequency Modulated Continuous Wave (FMCW) technology with rapid frequency ramps and real-time scanning capabilities [41]. A distinctive feature of this sensor is its ability to simultaneously measure long distances (up to 170 m), relative velocity, and the angle between two objects, making it well-suited for applications that require precise tracking of multiple spatial parameters.

The clustering algorithms allow one to identify, recognize, and track objects. A fusion of sensors, such as the camera and Lidar, provides greater performance, with accuracy and precision to meet AD—Driver Assistance and AV—Autonomous Vehicles.

To identify objects, the clustering algorithm must be applied to the point cloud, which was generated by the radar. The point cloud contains data about the coordinates of the points. After the algorithm is applied, we obtain the groups that can be directly associated with the objects.

## 5. Comparison Analysis of Cluster for Automotive Radar Systems

This section presents some simulations in the Driving Scenario Design tool from MATLAB, showing how the radar detects objects, such as pedestrians and cyclists, as well as stationary and moving vehicles. The results also show how the radar can detect several objects simultaneously, using MATLAB R2021a. Finally, a comparison among three algorithms is presented, focusing on K-Means, Mean Shift, and DBSCAN.

The tests were carried out with the 3D radar since its point clouds are sparse and have low resolution. This was conducted due to hardware limitations, as the use of a 4D radar would require more intensive computational resources. However, for a possible use of 4D radar, the algorithm that must be used for clustering is still DBSCAN, as it is a density-based algorithm.

To test the radar performance, we have to build up the test scenarios which comprise a radar component, vehicle, environment, and road users in a simulation framework based on the MATLAB Driving Scenario Design tool. The tests were carried out using MATLAB R2021a.

The test scenarios (1.1, 2.1, and 3.1) were simulated with a car composed of a radar, in a virtual environment, with dangerous weather characteristics and light traffic conditions on a highway. These test scenarios aim to verify how the radar detects a truck by applying different algorithms (K-Means, Mean Shift, and DBSCAN) to point cloud data generated from the detection.

The test scenarios (1.2, 2.2, and 3.2) were simulated with a car composed of a radar, in a virtual environment with dangerous weather characteristics and light traffic conditions on a highway. These test scenarios aim to verify how the radar detects a car by applying different algorithms (K-Means, Mean Shift, and DBSCAN) to the point cloud data generated from the detection.

The test scenarios (1.3, 2.3, and 3.3) were simulated with a car composed of a radar, in a virtual environment with dangerous weather characteristics and light traffic conditions on a highway. These test scenarios aim to verify how the radar detects a motorcyclist by applying different algorithms (K-Means, Mean Shift, and DBSCAN) to the point cloud data generated from the detection.

The test scenarios (1.4, 2.4, and 3.4) were simulated with a car composed of a radar, in a virtual environment with sunny meteorological characteristics and light traffic conditions in urban areas. These test scenarios aim to verify how the radar detects a pedestrian by applying different algorithms K-Means, Mean Shift, and DBSCAN) to the point cloud data generated from the detection.

The test scenarios (1.5, 2.5, and 3.5) were simulated with a car composed of a radar, in a virtual environment with sunny meteorological characteristics and light traffic conditions in urban areas. These test scenarios aim to verify how the radar detects a pedestrian by applying different algorithms (K-Means, Mean Shift, and DBSCAN) to the point cloud data generated from the detection.

Finally, a comparison among three algorithms is presented, focusing on K-Means, Mean Shift, and DBSCAN. The test scenarios are performed with trucks, cars, motorcyclists, pedestrians, and cyclists. Although we conducted a review of 10 clustering algorithms, we only analyze three clustering algorithms, K-Means, Mean Shift, and DBSCAN, as these algorithms’ characteristics are more similar to a distribution of radar-generated point clouds.

Only three clustering algorithms were chosen and analyzed, K-Means, Mean Shift, and DBSCAN, which have characteristics more similar to a distribution of point clouds generated by radar. The choice is also based on the process that each algorithm used. To perform the grouping, for the mathematical process that each algorithm uses and also the computational time that each algorithm takes to perform the grouping—that is, the greater the mathematical complexity of the algorithm, the greater the computational execution time, considering that we are applying in radar systems for autonomous vehicles and ADAS functions—the computational time is an important factor. This article presents the process and mathematics involved in each algorithm, so that the reader can read the characteristics of each algorithm in addition to also presenting a comparative table with the advantages and efficiency of using each algorithm.

The mention of the importance of the radar system in adverse weather conditions is to reinforce the importance of the automotive radar system in autonomous vehicles. Therefore, no tests have yet been carried out to present in this article, but we are working to carry out these tests together with the Radar ECU.

Table 3 describes test scenarios using the K-Means, Mean Shift, and DBSCAN algorithms, with a truck, car, motorcyclist, pedestrian, and cyclist, although we carried out a review of 10 clustering algorithms.

The simulations help to describe how the radar can recognize objects using the point clouds generated and how the clustering algorithms work using data from a radar system.

**Figure 16:**
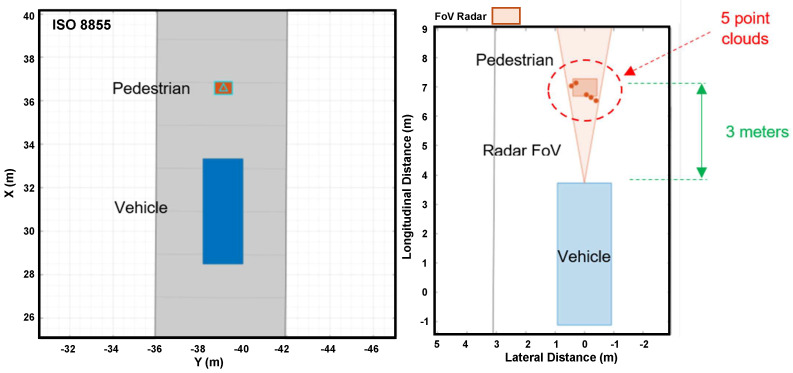
Radar detecting pedestrians in Driving Scenario Design.

Figure 16 shows how the simulated radar on a vehicle detects a pedestrian. On the left side, there is a top view of the vehicle and pedestrian moving, respectively, in longitudinal and lateral directions. The figure (on the right) shows the instant when the radar identifies the pedestrian, who is three meters forward, through five-point clouds.

**Figure 17:**
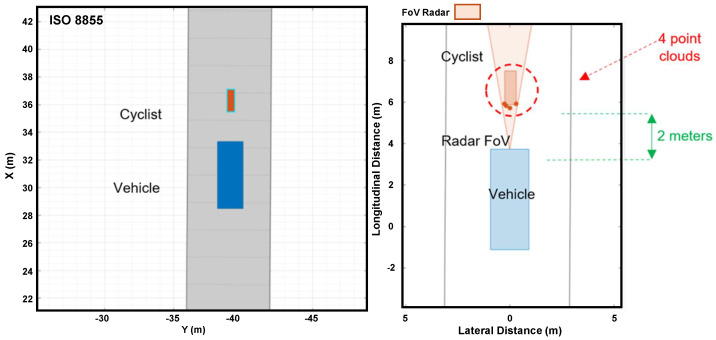
Radar detecting cyclist in Driving Scenario Design.

Figure 17 shows how the simulated radar on a vehicle detects a cyclist. On the left side, there is a top view of the vehicle and cyclist moving, respectively, in longitudinal and lateral directions. On the right side, the figure shows the instant when the radar identifies the cyclist, two meters forward, through four-point clouds.

**Figure 18:**
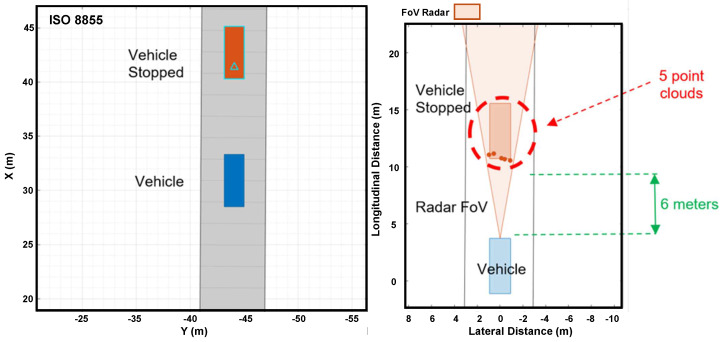
Radar detecting a stopped vehicle in Driving Scenario Design.

Figure 18 shows how the simulated radar on a vehicle detects a stationary vehicle. On the left side, there is a top view of the stationary vehicle in longitudinal and lateral directions. On the right side, the figure shows the instant when the radar identifies the stationary vehicle, six meters forward, through five-point clouds.

**Figure 19:**
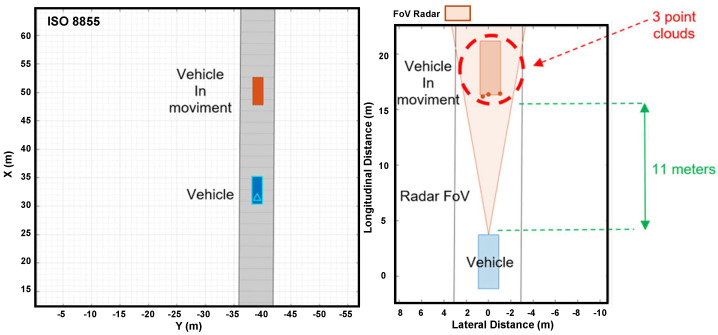
Radar detecting a moving vehicle in Driving Scenario Design.

Figure 19 shows how the simulated radar on a vehicle detects a vehicle in motion. On the left side, the figure shows a top view of the vehicle in motion in longitudinal and lateral directions. On the right side, the figure shows the instant when the radar identifies the stationary vehicle, three meters forward, through five-point clouds.

**Figure 20:**
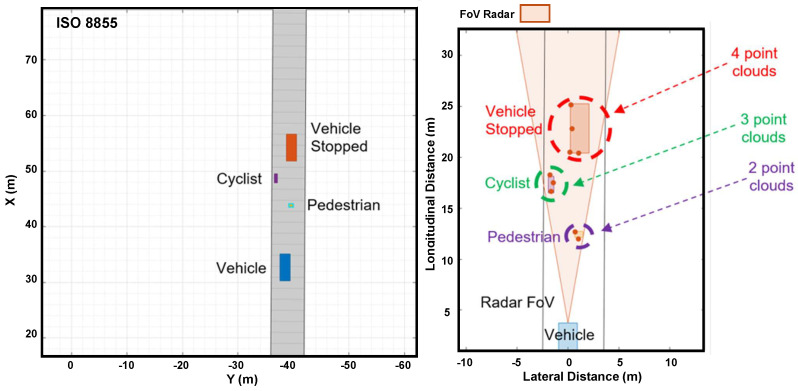
Radar detecting many objects in Driving Scenario Design.

Figure 20 shows how the radar simultaneously detects a stationary vehicle, a cyclist, and a pedestrian. As the vehicle approaches the objects, the radar detects all the points.

**Figure 21:**
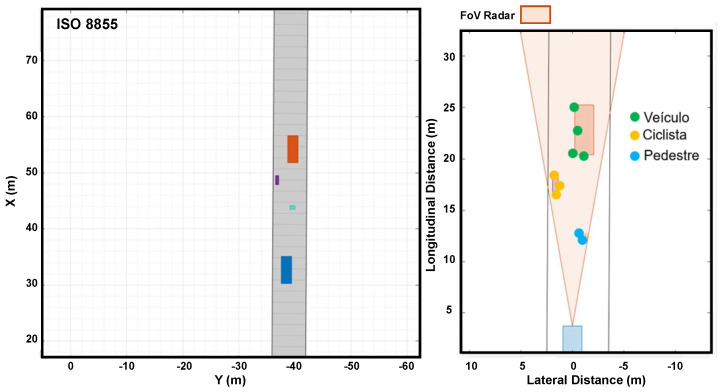
DBSCAN recognizing many objects.

The DBSCAN algorithm recognizes points in the presence of multiple objects. As the density of points is higher, the DBSCAN algorithm easily recognizes the groups of points. This facilitates the classification of these objects later, as shown Figure 21.

As the object is further from the radar, fewer points can be captured. For example, when a truck is far away, the radar only captures 5 points, but as the truck approaches, the radar begins to capture more points, such as 7 or 8.

The point clouds generated by the radar are composed of several points. By applying the cluster algorithm with the best performance, it is possible to distinguish objects accurately, even if the identified object has few points, as in addition to information such as directions, we can have information such as speed, azimuth angle, and depth. This allows us to have a more accurate identification.

The performance among the algorithms was determined by a quantitative analysis using the criteria determined by safety government and non-government agencies, such as EURONCAP and Latin NCAP, in documents referring to Autonomous Driving and automotive radar [42], such as Euro NCAP Roadmap 2025 IN SEARCH OF VISION ZERO and the Global Vehicle Goal Specification TB 025 [43]. The formulated metrics are as follows:

(a) Distance at which the algorithm identifies and recognizes objects.
(b) The actual distance of the objects; the further away the objects, the better.
(c) Speed of objects.

Soon after, just obtain the point clouds and apply the desired algorithm. These steps were for application in a simulation scenario in MATLAB very close to the real one, but to apply the algorithm in a point cloud scan of a real radar that is stored as a collection of 2D or 3D points depending on the generation of the radar, it is worth mentioning that the more modern, the better the resolution and quality of the points captured by the radar. As explained in the topic about radars, which contains the coordinates of objects around a vehicle, it is enough to have a “. mat” and apply it in the algorithm; see the example.

Load the x, y, or x, y, z coordinates of the objects, then define the area of interest. If you want to visualize the graph in 2D, and if you want to circle the objects of interest, apply the desired algorithm.

This is how clustering algorithms can be applied in radar systems for object classification and recognition. After this step of manual recognition by circling the objects, it is enough to recognize the patterns that the points generated by the radar form for each object and do a training of AI Machine Learning, neural networks such as YOLO v3, train the network to recognize these point patterns, and associate them with the objects so that the radar itself recognizes the objects starting from the clustering and the points generated by the radar. Thus, we have a tool that brings security and reliability to the use of object recognition for autonomous vehicles.

The results obtained in the simulations within the driving scenario tool are presented in the following tables, where simulations were performed for the K-Means, Mean Shift, and DBSCAN algorithms. Table 4, Table 5 and Table 6 presents the performance of previously mentioned algorithms. Similarly, Table 7 highlights the best-case performance scenario.

**Figure 22:**
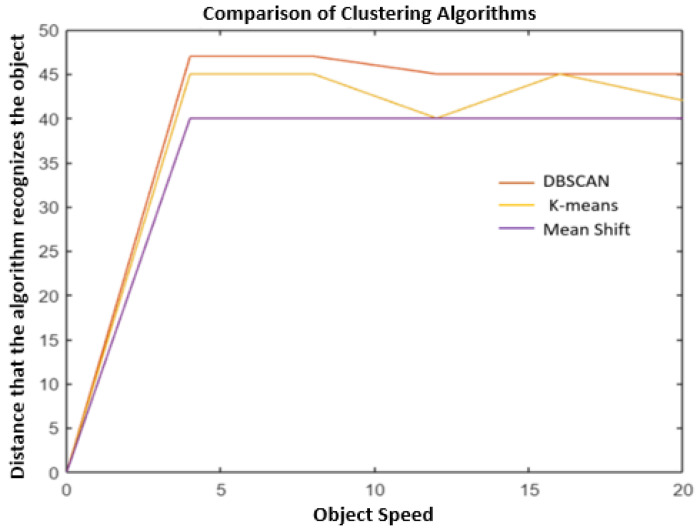
Comparing clustering algorithm.

Figure 22 presents the comparative analysis of the best performance of each algorithm. The scenario of the best performance of the three algorithms was with the object stationary, and the vehicles with the radars at 20 km/h.

Observing the graphs and results presented in the tables, the algorithm with the best performance in object recognition according to the evaluation statistics is DBSCAN, as presented in the theoretical foundation. As it is a density algorithm, it is a very successful algorithm for several applications, including in automotive radar systems for object recognition.

**Figure 23:**
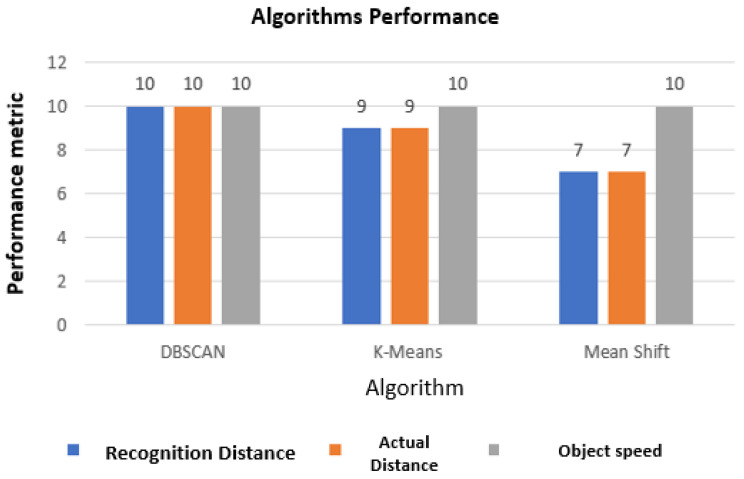
Performance of algorithms in tests.

The calculation of the scores described in Figure 23 below takes into account three factors:

The first is the distance that the algorithm can classify the object where the score varies from 0 to 10, where 0 means that the distance that the algorithm calculated is closest to the test vehicle, and 10 is how much the value returned by clustering is closest, far from the test vehicle. That is, the further away the algorithm recognizes the object, the better.

The distance considered to calculate the scores is 50 m, and as these are autonomous vehicles on the highway, this is a considerable distance for decision-making in the case of ADAS functions.

The second metric is the actual distance to the object, compared to the distance provided by the algorithm, where 0 means that the distance values are very different, and 10 means that the distance calculated by the algorithm is very close to the actual distance to the object. The distance considered to calculate the score is 50 m.

The third metric is the speed of the object when compared with the speed returned by the algorithm, where 0 means that the real speeds and those calculated by the algorithm are very different, and 10 means that the actual distances of the object and the distance calculated in the cluster are very close with tolerance below 5The relative speeds considered to calculate the score were 20, 40, and 60 km/h. As an example, the test vehicle was running at 100 km/h while other vehicles were at 120, 140, and 160 km/h.

Figure 23 shows the performance of the algorithms based on the scores (0 to 10) assigned to each metric.

## 6. Conclusions

In this study, we thoroughly evaluated the suitability of various clustering algorithms for automotive radar systems used in perception systems for ADAS and AVs. Our analysis encompassed a diverse set of algorithms, including Affinity Propagation, Hierarchical Clustering, DBSCAN, BIRCH, K-Means, Mini-Batch K-Means, OPTICS, Mean Shift, Spectral Clustering, and Mixture of Gaussians. The performance indicators used in our evaluation highlighted the particular strengths of K-Means, Mean Shift, and DBSCAN, demonstrating their efficacy in identifying, recognizing, and tracking objects through radar-based point cloud data.

Our findings emphasize the critical role of radar in perception systems, given its robustness under various weather conditions and its ability to utilize electromagnetic waves for object detection and classification. The integration of point cloud technology with clustering algorithms allows for precise definition and association of object shapes within the vehicle’s environment, contributing significantly to the reliability and robustness of navigation systems.

Moreover, this study underscores the importance of radar selection, as the choice of radar sensor considerably influences the performance of object recognition methods. The results indicate that for effective implementation of ADASs and autonomous vehicle technologies, it is essential to match the right clustering algorithm with the appropriate radar sensor to achieve optimal performance.

In conclusion, the insights gained from this evaluation can guide the development of more advanced and reliable perception systems, enhancing the efficiency and safety of autonomous technologies. Future work could explore the integration of these clustering algorithms with multi-sensor data-fusion techniques to further improve the robustness and accuracy of object detection and tracking in complex driving scenarios.

### Future Works

Building on the insights gained from this study, several avenues for future work are proposed to advance the development and implementation of perception systems for autonomous vehicles and driving assistance technologies:

- Multi-Sensor Data Fusion;
- Real-Time Processing Optimization;
- Machine Learning and AI Integration;
- Extended Field Testing;
- Adaptive Clustering Techniques;
- Collaboration with Industry Partners;
- Safety and Redundancy Mechanisms;
- Regulatory and Standardization Efforts.

## Figures and Tables

**Table 1:** Advantages and limitations among investigated Cluster Algorithms Part 1.

Algorithm	Advantage	Limitation
Affinity Propagation	Set number of clusters: No. Similarity principles. Used in matrix format. Base equation: $a\left(k,k\right)\leftarrow \sum_{i^{\prime }\;\mathrm{such}\;\mathrm{that}\;i\neq k}\max\left\{0,r\left(i^{\prime },k\right)\right\}$	The high complexity of the algorithm ends up making it slower than the others, Due to the construction of several matrices, the algorithm takes up a lot of memory algorithm
Hierarchical Clustering	Set number of clusters: No. Similarity principles works well in any situation points distribution. Base equation: $$\begin{array}{c} L\left(r,s\right)=\min\left(D\left(x_{ri},x_{si}\right)\right)\\ L\left(r,s\right)=\max\left(D\left(x_{ri},x_{si}\right)\right)\\ L\left(r,s\right)=\frac{1}{n_{r}n_{s}}\sum_{i=1}^{n_{r}}\sum_{j=1}^{n_{s}}D\left(x_{ri},x_{si}\right) \end{array}$$	Complexity is not linear $O\left(n^{3}\right)$
BIRCH	Set number of clusters: No. Hierarchical grouping. Similarity principles. Large datasets. Base equation: $x_{0}=\frac{1}{n}\sum_{i=1}^{n}x_{i}$	It only processes metric attributes, so it is only possible to apply if the values can be represented in space Euclidean, in this way no categorical attribute must be present.
DBSCAN	Set number of clusters: No. Connection by density applicable in different types of distribution of dice. Base equation: $N_{\in }\left(p\right)=\left\{q\;\mathrm{em}\;D|\mathrm{dist}\left(p,q\right)<\varepsilon \right\}$	It does not work as well when the data density is variable.
K-Means	Set number of clusters: Yes. Centroid models and similarities. Easy implementation. Base equation: $F_{S}\left(P\right)=\sum_{i=1}^{k}\sum_{x_{j}\in C_{i}}D\left(x_{j},y_{i}\right)$ $i^{*}=\underset{i=1,2,\ldots ,k}{\mathrm{argmin}}{∥x_{j}-y_{i}∥}_{2}^{2}$	It needs to estimate the number of cluster, in addition to being slow for large sets of data, and be able to converge to local minima.
Affinity Propagation	Set number of clusters: No. Similarity principles used in matrix format. Base equation: $a\left(k,k\right)\leftarrow \sum_{i^{\prime }\;\mathrm{such}\;\mathrm{that}\;i\neq k}\max\left\{0,r\left(i^{\prime },k\right)\right\}$	The high complexity of the algorithm ends up making it slower than the others. Due to the construction of several matrices, the algorithm takes up a lot of memory algorithm.
Hierarchical Clustering	Set number of clusters: No. Similarity principles work well in any situation points distribution. Base equation: $$\begin{array}{c} L\left(r,s\right)=\min\left(D\left(x_{ri},x_{si}\right)\right)\\ L\left(r,s\right)=\max\left(D\left(x_{ri},x_{si}\right)\right)\\ L\left(r,s\right)=\frac{1}{n_{r}n_{s}}\sum_{i=1}^{n_{r}}\sum_{j=1}^{n_{s}}D\left(x_{ri},x_{si}\right) \end{array}$$	Complexity is not linear. $O\left(n^{3}\right)$
BIRCH	Set number of clusters: No. Hierarchical grouping similarity principles. Large datasets. Base equation: $x_{0}=\frac{1}{n}\sum_{i=1}^{n}x_{i}$	It only processes metric attributes, so it is only possible to apply if the values can be represented in space Euclidean, in this way no categorical attribute must be present.
DBSCAN	Set number of clusters: No. Connection by density applicable in different types of distribution of dice. Base equation: $N_{\in }\left(p\right)=\left\{q\;\mathrm{em}\;D|\mathrm{dist}\left(p,q\right)<\varepsilon \right\}$	It does not work as well when the data density is variable.
K-Means	Set number of clusters: Yes. Centroid models and similarities. Easy implementation. Base equation: $F_{S}\left(P\right)=\sum_{i=1}^{k}\sum_{x_{j}\in C_{i}}D\left(x_{j},y_{i}\right)$ $i^{*}=\underset{i=1,2,\ldots ,k}{\mathrm{argmin}}{∥x_{j}-y_{i}∥}_{2}^{2}$	It needs to estimate the number of cluster, in addition to being slow for large sets of data, and be able to converge to local minima
Mini-Batch K-Means	Set number of clusters: Yes Centroid models and similarities. Easy implementation. Large number of clusters and data. Base equation: $F_{S}\left(P\right)=\sum_{i=1}^{k}\sum_{x_{j}\in C_{i}}D\left(x_{j},y_{i}\right)$ $i^{*}=\underset{i=1,2,\ldots ,k}{\mathrm{argmin}}{∥x_{j}-y_{i}∥}_{2}^{2}$	Not as efficient for small groups of data.
Mean Shift	Set number of clusters: No. Connection by density centroid models. Base equation: $y_{i}=\frac{1}{n_{i}}\sum_{x_{j}\in C_{i}}x_{j}$	Selecting the size of window/radius “r” may not be trivial; that is, if the parameters not defined with a value close to the ideal, the clusters do not come out like expected.

**Table 2:** Advantages and limitations among investigated Cluster Algorithms Part 2.

Algorithm	Advantages	Limitations
OPTICS	Set number of clusters: No. Connection by d accessibility chart. Base equation: dist(p,q)<ε	The time complexity of algorithm is $O\left(n^{2}\right)$ For databases with thousands of data, dimensionality, makes the points become sparse and obtain lost dense regions that characterize groups.
Spectral Clustering	Set number of clusters: No. Applicable for small sets. Used in matrix format graph partitioning. Base equation: $$D_{ij}=\left\{\begin{array}{c} d_{i},i=j\\ 0,i\neq j \end{array}\right.$$ L=D−A	Not recommended for large datasets. Since they are not scalars, the construction of matrices and calculation of eigenvectors and eigenvalues can be computationally intensive.
Mixture of Gaussians	Set number of clusters: No. Gaussian Mixture Mixing probability π Dimension ${ℜ}^{2}$ Base equation: $$\begin{array}{c} N\left(x|\mu ,\Sigma \right)=\frac{1}{{\left(2\pi \right)}^{\frac{D}{2}}{|\sum |}^{\frac{1}{2}}}\\ {}*\exp\left(-\frac{1}{2}{\left(x-\mu \right)}^{T}\Sigma^{-1}\left(x-\mu \right)\right) \end{array}$$ $$\sum_{k=1}^{K}\pi_{k}=1$$	Slow convergence. Converge to the optimal location only.

**Table 3:** This is a wide table describing test scenarios using the K-Means, Mean Shift, and DBSCAN algorithms for object recognition.

Test Scenario	Truck	Car	Motorcycle	Pedestrian	Cyclist
Algorithm K-Means	Scenario 1.1	Scenario 1.2	Scenario 1.3	Scenario 1.4	Scenario 1.5
Algorithm Mean Shift	Scenario 2.1	Scenario 2.2	Scenario 2.3	Scenario 2.4	Scenario 2.5
Algorithm DBSCAN	Scenario 3.1	Scenario 3.2	Scenario 3.3	Scenario 3.4	Scenario 3.5

**Table 4:** Test results obtained from K-Means algorithm.

Vehicle Speed	Metric	Scenario 1.1	Scenario 1.2	Scenario 1.3	Scenario 1.4	Scenario 1.5
20 km/h	Metric A	Distance 45 m	Distance 45 m	Distance 40 m	Distance 45 m	Distance 45 m
	Metric B	Distance 45 m	Distance 45 m	Distance 40 m	Distance 45 m	Distance 45 m
	Metric C	Stopped	Stopped	Stopped	Stopped	Stopped
40 km/h	Metric A	Distance 40 m	Distance 40 m	Distance 40 m	Distance 45 m	Distance 42 m
	Metric B	Distance 40 m	Distance 40 m	Distance 40 m	Distance 45 m	Distance 42 m
	Metric C	40 km/h	40 km/h	40 km/h	02 km/h	20 km/h
60 km/h	Metric A	Distance 40 m	Distance 40 m	Distance 40 m	Distance 45 m	Distance 40 m
	Metric B	Distance 40 m	Distance 40 m	Distance 40 m	Distance 45 m	Distance 40 m
	Metric C	60 km/h	60 km/h	60 km/h	04 km/h	30 km/h

**Table 5:** Test results obtained from Mean Shift algorithm.

Vehicle Speed	Metric	Scenario 2.1	Scenario 2.2	Scenario 2.3	Scenario 2.4	Scenario 2.5
20 km/h	Metric A	Distance 40 m	Distance 40 m	Distance 40 m	Distance 40 m	Distance 40 m
	Metric B	Distance 40 m	Distance 40 m	Distance 40 m	Distance 40 m	Distance 40 m
	Metric C	Stopped	Stopped	Stopped	Stopped	Stopped
40 km/h	Metric A	Distance 40 m	Distance 40 m	Distance 40 m	Distance 45 m	Distance 42 m
	Metric B	Distance 40 m	Distance 40 m	Distance 40 m	Distance 45 m	Distance 42 m
	Metric C	40 km/h	40 km/h	40 km/h	02 km/h	40 km/h
60 km/h	Metric A	Distance 40 m	Distance 40 m	Distance 40 m	Distance 45 m	Distance 40 m
	Metric B	Distance 40 m	Distance 40 m	Distance 40 m	Distance 45 m	Distance 40 m
	Metric C	60 km/h	60 km/h	60 km/h	40 km/h	30 km/h

**Table 6:** Test results obtained from DBSCAN algorithm.

Vehicle Speed	Metric	Scenario 3.1	Scenario 3.2	Scenario 3.3	Scenario 3.4	Scenario 3.5
20 km/h	Metric A	Distance 47 m	Distance 47 m	Distance 45 m	Distance 45 m	Distance 45 m
	Metric B	Distance 47 m	Distance 47 m	Distance 45 m	Distance 45 m	Distance 45 m
	Metric C	Stopped	Stopped	Stopped	Stopped	Stopped
40 km/h	Metric A	Distance 47 m	Distance 47 m	Distance 45 m	Distance 45 m	Distance 45 m
	Metric B	Distance 47 m	Distance 47 m	Distance 45 m	Distance 45 m	Distance 45 m
	Metric C	40 km/h	40 km/h	40 km/h	02 km/h	40 km/h
60 km/h	Metric A	Distance 47 m	Distance 47 m	Distance 45 m	Distance 45 m	Distance 45 m
	Metric B	Distance 47 m	Distance 47 m	Distance 45 m	Distance 45 m	Distance 45 m
	Metric C	60 km/h	60 km/h	60 km/h	40 km/h	30 km/h

**Table 7:** Best case performance of each algorithm.

Vehicle Speed	Metric	Scenarios 1.1 - 2.1 - 3.1	Scenarios 1.2 - 2.2 - 3.2	Scenarios 1.3 - 2.3 - 3.3	Scenarios 1.4 - 2.4 - 3.4	Scenarios 1.5 - 2.5 - 3.5
20 km/h DBSCAN	Metric A	Distance 47 m	Distance 47 m	Distance 45 m	Distance 45 m	Distance 45 m
	Metric B	Distance 47 m	Distance 47 m	Distance 45 m	Distance 45 m	Distance 45 m
	Metric C	Stopped	Stopped	Stopped	Stopped	Stopped
20 km/h K-Mean	Metric A	Distance 45 m	Distance 45 m	Distance 40 m	Distance 45 m	Distance 42 m
	Metric B	Distance 45 m	Distance 45 m	Distance 40 m	Distance 45 m	Distance 42 m
	Metric C	Stopped	Stopped	Stopped	Stopped	Stopped
20 km/h Mean Shift	Metric A	Distance 40 m	Distance 40 m	Distance 40 m	Distance 40 m	Distance 40 m
	Metric B	Distance 40 m	Distance 40 m	Distance 40 m	Distance 40 m	Distance 40 m
	Metric C	Stopped	Stopped	Stopped	Stopped	Stopped

## Data Availability

Data is contained within the article.

## Abbreviations

The abbreviations mentioned in the topic below are used in this manuscript:

ADAS	Advanced Driver Assistance System
AD	Autonomous Driving
DBSCAN	Density-Based Spatial Clustering of Noise Applications
GMMs	Gaussian Mixture Models
FMCW	Frequency-modulated Continuous Wave
MMIC	Monolithic Microwave-Integrated Circuit
RADAR	Radio Detection and Ranging
LIDAR	Light Detection and Ranging
BIRCH	Balanced Iterative Reducing and Clustering using Hierarchies
OPTICS	Ordering Points to Identify the Clustering Structure
